# Associations Between Muscle Thickness, Motor Function, and Echo Intensity in Community-Dwelling Older Japanese Men and Women

**DOI:** 10.7759/cureus.76651

**Published:** 2024-12-30

**Authors:** Ken Nishihara, Hisashi Kawai, Manami Ejiri, Keigo Imamura, Shuichi Obuchi

**Affiliations:** 1 Physical Therapy, Saitama Prefectural University, Koshigaya, JPN; 2 Research Team for Human Care, Tokyo Metropolitan Institute for Geriatrics and Gerontology, Tokyo, JPN

**Keywords:** community-dwelling older person, echo intensity, motor function, muscle thickness, ultrasound

## Abstract

Purpose

Muscle atrophy progresses with age. The motor function may be estimated by measuring the muscle mass; however, if muscle quality deteriorates due to an increase in connective tissue within the muscle, a decline in motor function may be missed by measuring muscle mass alone. Therefore, it is important to understand the relationship between muscle mass, muscle quality, and motor function. This study aimed to clarify how changes in muscle thickness, measured using ultrasound imaging, in older people are related to motor function and echo intensity.

Patients and methods

The thickness and echo intensity of the four quadriceps muscles were measured using an ultrasound imaging device in 110 community-dwelling older individuals. Correlations between muscle thickness, motor functions such as walking and muscle strength, and echo intensity were analyzed in each sex. Partial correlation analysis was conducted using age as a control variable.

Results

A significant correlation was observed between muscle thickness and motor function in both men and women. There was no significant negative correlation between muscle thickness and echo intensity of the same muscle in men, except between each muscle thickness and the vastus medialis (p < 0.001 or p = 0.007), between the sum of the four muscle thicknesses and vastus medialis thickness (p = 0.02), and between the vastus medialis thickness and echo intensity (p < 0.006). In women, a significant correlation was observed for all muscles. Partial correlation analysis revealed a similarly significant correlation between muscle thickness and echo intensity in men and women.

Conclusion

We observed sex differences in the relationship between muscle thickness and echo intensity. This suggests that, even after adjusting for age-related factors, women may show more pronounced changes in muscle quality than men, with increased echo intensity due to increased intramuscular connective tissue with muscle atrophy.

## Introduction

With aging, motor functions - such as balance and the ability to walk - deteriorate. Psychological changes include changes in levels of hormones and central nervous system neurotransmitter secretion, which decrease the motivation to be active and reduce physical activity [1,2]. As physical activity declines, motor function declines further, resulting in a decrease in the quality of life.

Decreased muscle strength is an important factor in motor function decline. Muscle weakness and a decline in motor function can influence each other, creating a vicious cycle. It is important to prevent the decline in motor function due to aging for as long as possible. Among motor functions, a decline in walking limits independence in the elderly. Mortality rates increase in those with slower walking speeds than those with faster walking speeds [3]. Furthermore, a decline in walking speed is related to a decrease in skeletal muscle mass, mainly in the lower body, due to aging [4].

Muscle weakness can be estimated from a decrease in muscle mass, which indicates muscle atrophy; thus, ultrasound imaging devices, which are safe and radiation-free, have been used to evaluate muscle mass using muscle thickness as a screening method [5-7]. However, it has been reported that muscle weakness in grip strength, quadriceps, and other muscles is more strongly associated with the decreased ability to perform activities of daily living and mortality than with decreased muscle mass, suggesting that muscle weakness may be overlooked by measuring muscle mass alone [8]. The reason for the limitation of muscle strength estimation by muscle mass is thought to be that the decrease in the neurological excitability necessary for exercise and the slowing of protein synthesis during muscle recovery cause an increase in the adipocyte content of intermuscular adipose tissue between muscle groups and muscle bundles, resulting in a decrease in exercise function without a change in muscle thickness [9]. Ultrasound imaging systems can measure echo intensity, which can be used to reflect changes in muscle quality due to the accumulation of intramuscular adipose tissue [10]. Investigating the characteristics of the relationship between muscle thickness, echo intensity, and motor function will provide a basis for understanding the effects of muscle mass loss on muscle quality and motor function loss and for interpreting the results of muscle thickness and echo intensity measurements.

In this study, we analyzed the relationship between muscle mass, motor function, and muscle quality in community-dwelling older men and women using the quadriceps muscle group, which is one of the most dominant muscles involved in walking and other activities.

## Materials and methods

Participants

The study included 623 community-dwelling people aged 65 years and older who participated in the "Otassha Study 2011 Cohort" 2022 survey. The participants were stratified by sex and age groups (60, 70, and 80 years and over) and classified into sarcopenic and non-sarcopenic based on the criteria of the Asian Working Group for Sarcopenia 2019 [11]. Sampling was performed to include approximately 20 people (at least 10 people with sarcopenia) in each stratified group. When a stratified group contained more than 20 people, the 1/2, 1/3, or 1/6 sampling was performed depending on the number of people to adjust the total number of participants to approximately 160. Consequently, 157 participants were selected, and invitations were mailed to additional measurement sessions for this study.

This study was approved by the Ethics Committee of the Tokyo Metropolitan Institute of Gerontology (approval no. R22-034) and the Ethics Committee of Saitama Prefectural University (approval no. 27055). The purpose and content of the study were explained to the participants, and measurements were started after obtaining their consent.

Measuring items

Measurement of muscle thickness: An ultrasound imaging system (Samsung MEDISON, Mysono U6, South Korea) was used with a linear probe set to B-Mode 8.8 MHz and a depth of focus of 2.5 cm. The participant was placed in the supine position, and the leg was on the same side as the knee. The extensor muscle strength measurement was imaged in the short-axis direction. Additional gel for the ultrasound was applied over the skin to avoid pressing the probe too hard on the skin. A depth of 5 cm was imaged from the skin to the surface of the bone, including the bone surface so that the participants' femurs could be seen. The muscle thickness was measured as described below.

**Figure 1:**
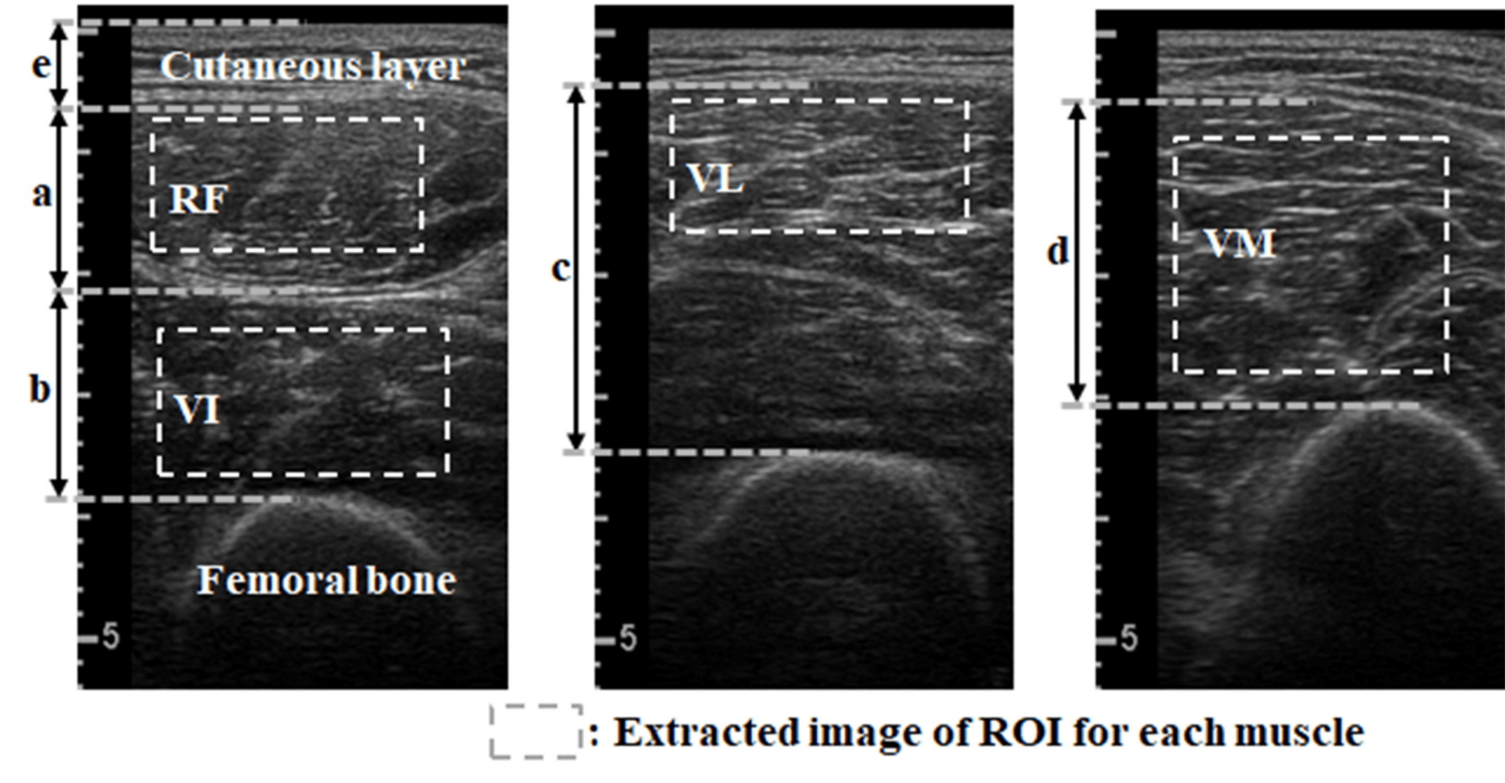
Ultrasound imaging of the quadriceps femoris to measure muscle and cutaneous thicknesses and echo intensities. The mean of grayscale values is considered the echo intensity. A: RF thickness; B: VI thickness; C: VL thickness; D: VM thickness; E: cutaneous thickness Abbreviations: RF, rectus femoris; ROI, region of interest; VI, vastus intermedius; VL, vastus lateralis; VM, vastus medialis

Rectus femoris (RF) and vastus intermedius (VI) thickness: The probe was placed at the center of the distance from the lateral epicondyle to the superior anterior iliac spine. The angle of the probe was fine-tuned so that the femoral surface at the back was the clearest and most vertical for imaging (Figure 1). The fascia was used as a reference from shallowest to thickest for the RF and VI thicknesses.

Vastus lateralis (VL) thickness, including the vastus medialis (VM) thickness (defined as VL thickness in this study): The probe was placed at the midpoint between the lateral epicondyle and the greater trochanter. The VL thickness extended from the shallow fascia to the femoral surface but included the VM near the femur.

VM thickness: The probe was placed at 30% of the distance from the lateral epicondyle to the greater trochanter. VM thickness was defined as the area from the shallow fascia to the femoral surface.

Sum of the four muscle (SFM) thicknesses: The thicknesses of the four quadriceps muscles described above were summed.

Skin thickness: Skin thickness was measured using images of the RF and VM as reference data. Skin thickness was defined as the area from the skin surface to the fascia of the RF.

Measurement of motor function

The following motor functions were measured in the Ottasha Study 2022 survey. The participant may not have participated in some measurements because of criteria based on vital signs or at their request. The participants wore heel-less shoes during the measurements. The measurements of motor function are described below.

Grip strength: The grip strength of the dominant hand (stronger hand) was measured with a grip dynamometer (As One, Osaka, Japan) while the participant was kept in a stable upright posture with both legs naturally open to prevent compensatory movement.

Knee extension strength: A knee extension muscle strength-measuring device (Isoforce GT610S; OG Giken, Okayama, Japan) with the measuring device fixed to a special frame was used. The knee extension torque was calculated by multiplying the greater of two measurements of the pressure value (unit: Newton (N)) of the measuring instrument at isometric maximum knee extension on the dominant leg by the distance (unit: meters (m)) from the lateral epicondyle of the knee by the measuring instrument sensor. The knee extension muscle strength was calculated.

Normal walking time: The time taken to walk a 5 m walking path with a 3 m spare path was measured. The instruction was standardized to "walk as you normally walk.”

Maximum walking time: This was measured the same way as the normal walking time, with the instruction "walk forward as fast as possible.”

Timed up and go test (TUG test): The participants were asked to stand up from a posture with their backs against the back of the chair, turn around at a 3 m marker, and sit down again. Smaller values were obtained for both measurements.

Measurement of echo intensity

The regions of interest were selected from the muscle areas of the ultrasound images. The images were imported using Adobe Photoshop CS6 (version 13.0; Adobe Systems, San Jose, CA) and converted to a black-and-white bitmap format. A grayscale value of 0 for black and 255 for white for each pixel in each image was averaged to obtain the echo intensity. The muscle strength was measured as described below.

Region of interest for RF and VM echo intensity: For each RF and VM, the largest square, excluding the fascia, was the region of interest.

Region of interest for VL echo intensity: The largest possible square of the VL was defined as the region of interest by identifying the fascia separating the VL.

Region of interest for VM echo intensity: The quadrangle within the VM was defined as the region of interest.

Statistical analysis

For all variables, normality was confirmed using the Shapiro-Wilk test. Differences between men and women were examined using the t-test. Pearson's correlation coefficient was calculated to determine the correlation between motor function, echo intensity, and muscle thickness. In addition, the partial correlation coefficients were calculated using age as a control variable. The correlation coefficients were interpreted as follows: absolute values of 0.2-0.4 indicated a slight correlation, values of 0.4-0.7 indicated a significant correlation, and values of 0.7-1.0 indicated a strong correlation. IBM SPSS Statistics for Windows (version 29.0.2.0; IBM Corp., Armonk, NY) was used for statistical analysis, and the significance level was set at 5%.

## Results

Participant characteristics

Finally, all 110 individuals participated in the additional sessions and were included in the analysis (age 74.3 ± 6.9 years (mean ± standard deviation), 46 men and 64 women; Table 1). Ten men and 25 women were determined to have sarcopenia, four men and four women had a history of stroke, 15 men and 14 women had a history of heart disease, and 12 men and five women had a history of diabetes. Regarding the basic characteristics of the participants, there was no significant difference in age between the men and women; however, the height, weight, and body mass index (BMI) were higher in men. Of the four muscle thicknesses measured, the VL and VM thickness were significantly greater in men. Regarding motor function, men showed significantly higher grip strength and knee extension muscle strength; however, there were no differences in the normal walking time, maximum walking time, or the TUG test. Regarding muscle brightness, women showed significantly higher RF, VL, and VM values.

**Table 1:** Subject characteristics by sex. Mean ± Standard deviation; *p < 0.05, **p < 0.01, †p < 0.001 Abbreviations: BMI, body mass index; QF, quadriceps femoris; RF, rectus femoris; TUG, timed up and go; VI, vastus intermedius; VL, vastus lateralis; VM, vastus medialis

Variables	Men (n = 46)	Women (n = 64)	p-value
Age (years)	74.4 ± 7.6	74.2 ± 6.4	0.857
Height (cm)	163.9 ± 7.0	151.5 ± 5.8	0.001^†^
Body mass (kg)	62.3 ± 11.0	49.0 ± 9.0	0.000^†^
BMI (kg/m^2^)	23.2 ± 3.5	21.3 ± 3.4	0.006**
Thickness of four muscles (mm)	85.8 ± 14.6	76.6 ± 17.0	0.004**
Thickness of RF (mm)	17.5 ± 3.0	16.3 ± 3.8	0.085
Thickness of VI (mm)	14.7 ± 3.3	13.6 ± 4.3	0.138
Thickness of VL (mm)	31.9 ± 6.2	27.8 ± 6.2	0.001*
Thickness of VM (mm)	21.8 ± 6.0	18.8 ± 5.8	0.012*
Cutaneous thickness (mm)	7.3 ± 2.6	10.6 ± 4.1	0.000†
Grasp strength (kg)	31.1 ± 6.2	17.9 ± 4.9	0.000†
Normal walk (s)	4.0 ± 0.8	3.8 ± 0.7	0.207
Fastest walk (s)	2.4 ± 0.5	2.6 ± 0.5	0.055
TUG test (s)	5.4 ± 1.4	5.4 ± 1.2	0.846
Knee extension strength (Nm)	99.4 ± 34.1	62.5 ± 22.3	0.000†
Echo intensity of RF	59.4 ± 8.7	64.8 ± 8.2	0.006**
Echo intensity of VI	45.2 ± 9.4	46.4 ± 13.4	0.597
Echo intensity of VL	59.8 ± 11.1	64.8 ± 8.2	0.007**
Echo intensity of VM	64.2 ± 10.3	71.0 ± 11.5	0.002**

Correlation between muscle thickness and motor function

**Figure 2:**
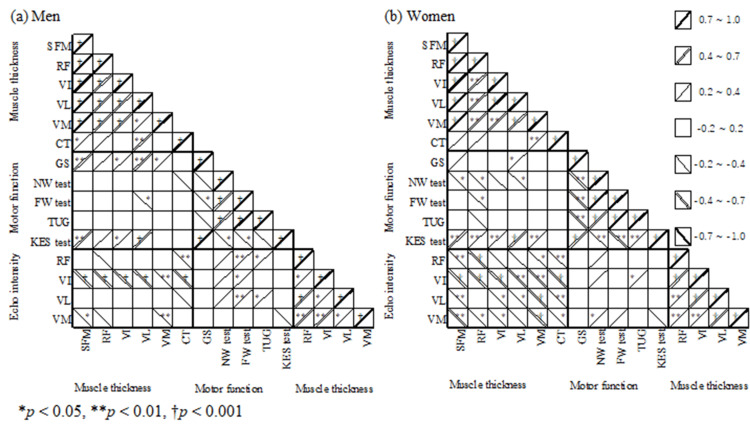
Heatmaps of the correlation coefficients between muscle thickness, physical functions, and echo intensities. Abbreviations: CT, cutaneous thickness; FW, fast walking; GS, grip strength; KES, knee extension strength; NW, normal walking; RF, rectus femoris; SFM, sum of the four muscles; TUG, timed up and go; VI, vastus intermedius, VL, vastus lateralis, VM, vastus medialis

In men, there was a significant correlation between grip strength and knee extension torque with respect to SFM (p = 0.001 and p = 0.003, respectively, Table 2, Figure 2; p values are not specified in this table). For individual muscles, there was a slight or significant correlation between grip strength and knee extension torque with individual muscles. In women, the SFM thickness was slightly correlated with the normal walking time (p = 0.033) and significantly correlated with knee extensor strength (p < 0.001). For individual muscles, there was a slight correlation with grip strength and normal and maximum walking times and a slight or significant correlation with knee extensor strength.

**Table 2:** Correlation coefficients between muscle thicknesses and measurements of physical functions and echo intensities. *p < 0.05, **p < 0.01, †p < 0.001 Abbreviations: CC, correlation coefficient; CT, cutaneous thickness; FW, fast walking; GS, grip strength; KES, knee extension strength; NW, normal walking; RF, rectus femoris, SFM, sum of the four muscles, TUG, timed up and go; VI, vastus intermedius, VL, vastus lateralis, VM, vastus medialis

		Muscle thickness					CT		Motor function						Echo intensity			
Sex	Muscle thickness	SFM	RF	VI	VL	VM			GS	NW test	FW test	TUG	KES test		RF	VI	VL	VM
Men	SFM	1.000	0.757**	0.808**	0.803**	0.771**	0.298*		0.462**	-0.100	-0.182	0.005	0.423**		-0.034	-0.683**	-0.022	-0.343*
	RF	0.757†	1.000	0.557†	0.515†	0.494†	0.214		0.256	-0.006	-0.009	0.113	0.223		-0.231	-0.551†	-0.101	-0.269
	VI	0.808†	0.557†	1.000	0.598†	0.509†	0.069		0.354*	-0.157	-0.285	-0.153	0.341*		0.017	-0.660†	0.022	-0.182
	VL	0.803†	0.515†	0.598†	1.000	0.327*	0.486**		0.450**	-0.187	-0.342*	-0.154	0.535†		-0.166	-0.602†	-0.203	-0.190
	VM	0.771†	0.494†	0.509†	0.327*	1.000	0.078		0.331*	0.039	0.073	0.198	0.175		0.193	-0.395**	0.193	-0.399**
	CT	0.298*	0.214	0.069	0.486**	0.078	1.000		0.073	-0.265	-0.060	0.038	0.038		-0.396**	-0.507†	-0.262	-0.197
Women	SFM	1.000	0.839†	0.852†	0.882†	0.789†	0.245		0.211	-0.272*	-0.173	-0.151	0.491†		-0.333**	-0.518†	-0.379**	-0.400**
	RF	0.839†	1.000	0.657†	0.672†	0.580†	0.244		0.155	-0.260*	-0.256*	-0.223	0.394**		-0.551†	-0.536†	-0.197	-0.270*
	VI	0.852†	0.657†	1.000	0.741†	0.505†	0.078		0.111	-0.207	-0.160	-0.106	0.395**		-0.221	-0.429†	-0.293*	-0.251*
	VL	0.882†	0.672†	0.741†	1.000	0.508†	0.117		0.273*	-0.258*	-0.155	-0.198	0.496†		-0.201	-0.423**	-0.294*	-0.246
	VM	0.789†	0.580†	0.505†	0.508†	1.000	0.397**		0.171	-0.187	-0.061	-0.021	0.342**		-0.252*	-0.401**	-0.445†	-0.540†
	CT	0.245	0.244	0.078	0.117	0.397**	1.000		0.073	-0.265	-0.060	0.038	0.038		-0.396**	-0.507†	-0.262	-0.197

**Figure 3:**
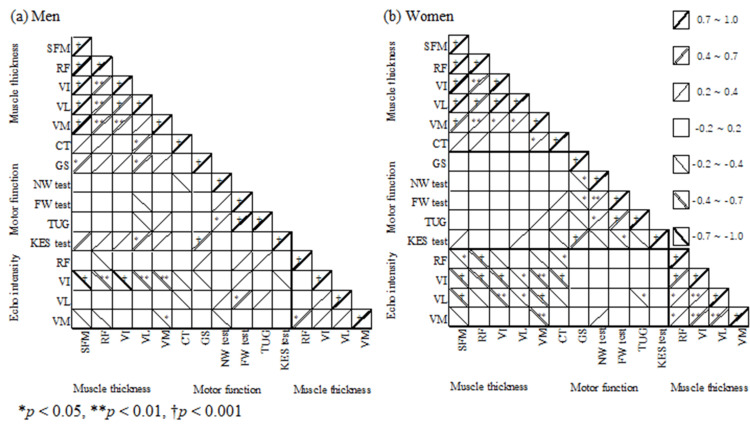
Heatmaps of the partial correlation coefficients with the limiting value of age between muscle thickness, physical functions, and echo intensities. Abbreviations: CT, cutaneous thickness; FW, fast walking; GS, grip strength; KES, knee extension strength; NW, normal walking; RF, rectus femoris; SFM, sum of the four muscles; TUG, timed up and go; VI, vastus intermedius, VL, vastus lateralis, VM, vastus medialis

In the partial correlation analysis with age as the limiting variable, there was a slight correlation between grip strength and SFM thickness in men (p = 0.027, Table 3, Figure 3). Among individual muscles, there was a significant correlation between grip strength and knee extension strength in the VL (p = 0.015 and p = 0.01, respectively). For women, there were no significant correlations.

**Table 3:** Partial correlation coefficients with a limit value of the age between muscle thicknesses and measurements of physical functions and echo intensities. *p < 0.05, **p < 0.01, †p < 0.001 Abbreviations: CC, correlation coefficient; CT, cutaneous thickness; FW, fast walking; GS, grip strength; KES, knee extension strength; NW, normal walking; RF, rectus femoris, SFM, sum of the four muscles, TUG, timed up and go; VI, vastus intermedius, VL, vastus lateralis, VM, vastus medialis

		Muscle thickness					CT		Motor function						Echo intensity			
Sex	Muscle thickness	SFM	RF	VI	VL	VM			GS	NW test	FW test	TUG	KES test		RF	VI	VL	VM
Men	SFM	1.000	0.834†	0.867†	0.813†	0.792†	0.349		0.451*	0.038	-0.135	0.070	0.402		-0.007	-0.782†	-0.103	-0.247
	RF	0.834†	1.000	0.636**	0.575**	0.649**	0.210		0.271	0.052	-0.016	0.085	0.200		-0.235	-0.628**	-0.185	-0.252
	VI	0.867†	0.636**	1.000	0.673†	0.611**	0.188		0.365	-0.027	-0.142	0.007	0.319		0.007	-0.761†	-0.008	-0.191
	VL	0.813†	0.575**	0.673†	1.000	0.357	0.487		0.491*	-0.095	-0.380	-0.224	0.516*		-0.052	-0.637**	-0.175	0.022
	VM	0.792†	0.649**	0.611**	0.357	1.000	0.178		0.303	0.196	0.146	0.382	0.214		0.175	-0.576**	0.022	-0.429*
	CT	0.349	0.210	0.188	0.487	0.178	1.000		0.034	-0.238	-0.175	-0.127	0.177		-0.153	-0.375	-0.217	0.063
Women	SFM	1.000	0.738†	0.805†	0.866†	0.679†	0.225		0.067	-0.065	0.081	0.044	0.235		-0.384*	-0.656†	-0.561†	-0.294
	RF	0.738†	1.000	0.484**	0.527†	0.399**	0.278		0.030	-0.028	-0.021	-0.104	0.094		-0.652†	-0.686†	-0.211	-0.141
	VI	0.805†	0.484**	1.000	0.721†	0.309*	0.047		-0.069	0.026	0.038	0.020	0.184		-0.240	-0.554†	-0.478**	-0.189
	VL	0.866†	0.527†	0.721†	1.000	0.353*	0.018		0.100	-0.152	0.029	-0.042	0.304		-0.152	-0.383*	-0.383*	-0.117
	VM	0.679†	0.399	0.309	0.353	1.000	0.390		0.109	-0.009	0.181	0.231	0.103		-0.269	-0.498**	-0.623†	-0.454**
	CT	0.225	0.278	0.047	0.018	0.39*	1.000		-0.144	0.098	0.270	0.252	-0.289		-0.352*	-0.581†	-0.287	-0.191

Correlation between muscle thickness and echo intensity

In men, there was a significant negative correlation with VI echo intensity (p < 0.001) and a slight negative correlation with VM echo intensity (p = 0.02, Table 2, Figure 2) with respect to SFM thickness. There was a significant negative correlation with the VI echo intensity for the RF, VM, and VL thicknesses (all p < 0.001, except VM p < 0.007) and a significant negative correlation with VM echo intensity for VM thickness (p = 0.006). In women, there was a negative correlation with each of the four echo intensities for SFM thickness (p < 0.001 or p = 0.001-0.008) and a slight or significant negative correlation with most echo intensities for each of the four muscle thicknesses (p < 0.001 or p = 0.001-0.049).

In the partial correlation analysis with age as the limiting variable, there was a strong negative correlation in men with a VI echo intensity for SFM thickness (p < 0.001, Table 3), a significant or strong negative correlation with the VI echo intensity for each of the four muscle thicknesses (p < 0.001 or p = 0.001-0.003), and a significant negative correlation with VM echo intensity for VM thickness (p < 0.037). In women, there was a slight negative correlation between RF echo intensity and SFM thickness (p = 0.012) and a significant negative correlation between the intermediate and VL echo intensities (both p < 0.001). There was a slight or significant negative correlation with most echo intensities for the four muscle thicknesses (p < 0.001 or p = 0.001-0.012).

## Discussion

This study examined the correlation between thigh muscle thickness, motor function, and echo intensity in community-dwelling older adults by comparing men and women. The results of this study indicate that muscle thickness is correlated with motor function, mainly knee extension muscle strength, in both men and women (Table 2). The quadriceps muscle thickness measured in this study can be used to evaluate knee extension muscle strength. In the elderly, muscle mass and strength decline unless they engage in habitual exercise and proper nutrition. This decline is more pronounced in the muscle groups of the lower limbs than in the upper limbs [4]. Focusing on the decrease in muscle mass due to aging and disease, sarcopenia has been defined as the loss of muscle strength and other motor functions, with the main factors being aging, inactivity, disease, and malnutrition [12]. A high correlation between muscle thickness and knee extension muscle strength may reflect sarcopenia; however, it is now known that, even if muscle mass does not change much, it can still cause a decline in motor function and affect the quality of life, which is now defined as dynapenia [13]. The thickness of the RF and VM did not significantly correlate with knee extension muscle strength in men, which may reflect dynapenia.

The decrease in motor function, especially muscle strength, without a significant decrease in muscle mass, suggests an increase in connective tissue that does not contribute to muscle contraction and a decrease in muscle quality from the perspective of the muscles responsible for movement. In other words, even if a person's muscle mass has not changed significantly, it is important to note whether motor function has declined. In ultrasound images, the connective tissue accumulation increases the mean value of echo intensity in the regional image portion of the muscle [14].

There was no significant correlation between the echo intensity and knee extension muscle strength measured in this study in either men or women (Figure 2). Conversely, a systematic review of 51 papers reported that the echo intensity of the quadriceps mostly showed a moderate negative correlation with knee extension muscle strength and negative correlations with other echo intensity, such as grip strength, walking speed, standing movements (sit-to-stand), and TUG tests [15]. In the present study, the effect of increased connective tissue within the muscle on motor function was not significant, suggesting that older people could compensate for the changes in muscle quality. However, the significant correlation between muscle thickness and muscle strength, in addition to the significant negative correlation with echo intensity, suggests that, with the decline in muscle mass, muscle quality also changes. It should be noted that this phenomenon is particularly evident in women. Muscle mass, exercise function, and quality are expected to decline with age in the older population. However, the correlation between muscle thickness and echo intensity persisted even when age was excluded, and this relationship was more pronounced in women. In women, a decrease in muscle thickness, even when age-related changes are excluded, suggests a decline in motor function and an increase in echo intensity. Conversely, although men exhibit higher levels of muscle mass and strength than women, age-related progressive declines have been reported to be maintained more in women than in men [16,17]. This may be partly because the decrease in muscle thickness in women is maintained by increased connective tissue in the muscle.

A limitation of this study is that the results could be biased toward relatively healthy individuals because the participants decided to undergo additional measurement sessions for this cohort survey. Therefore, further studies that represent the general elderly population are needed to verify the study results. Moreover, although this study attempted to measure the muscle thickness of the four quadriceps, the fifth muscle, called the tensor vastus intermedius, was not included in this study [18]. The ultrasound image of the VL included the VM at a deeper layer, and in a few participants, the fascia that distinguishes the VL from the VM was unclear. Therefore, in this study, the VL included the VM thickness.

## Conclusions

A decline in muscle strength and other motor functions can occur when there is a decrease in muscle mass due to muscle atrophy, a decrease in muscle quality due to elevated connective tissue, or both. Muscle assessment and quantification are important for preventing or improving the decrease in motor function caused by muscle weakness. The findings of this study suggest that muscle thickness correlates mainly with muscle strength, such as grip strength and knee extension muscle strength, in older men and with activities of daily living, such as normal walking, and knee extension muscle strength in older women. Additionally, this study suggested that quadriceps muscle thickness correlated more frequently with the respective echo intensities in older women than in older men. More attention should be paid to changes in muscle quality during a decline in motor function in women. This study may help us consider the influence of muscle quality and sex when estimating and screening motor function based on muscle thickness.
